# A rapid and versatile method for the isolation, purification and cryogenic storage of Schwann cells from adult rodent nerves

**DOI:** 10.1038/srep31781

**Published:** 2016-08-23

**Authors:** Natalia D. Andersen, Shruthi Srinivas, Gonzalo Piñero, Paula V. Monje

**Affiliations:** 1 The Miami Project to Cure Paralysis and Department of Neurological Surgery, University of Miami Miller School of Medicine, Miami, Florida 33136, USA; 2 Universidad de Buenos Aires, CONICET, Instituto de Química y Fisicoquímica Biológicas (IQUIFIB), Buenos Aires, Argentina

## Abstract

We herein developed a protocol for the rapid procurement of adult nerve-derived Schwann cells (SCs) that was optimized to implement an immediate enzymatic dissociation of fresh nerve tissue while maintaining high cell viability, improving yields and minimizing fibroblast and myelin contamination. This protocol introduces: (1) an efficient method for enzymatic cell release immediately after removal of the epineurium and extensive teasing of the nerve fibers; (2) an adaptable drop-plating method for selective cell attachment, removal of myelin debris, and expansion of the initial SC population in chemically defined medium; (3) a magnetic-activated cell sorting purification protocol for rapid and effective fibroblast elimination; and (4) an optional step of cryopreservation for the storage of the excess of cells. Highly proliferative SC cultures devoid of myelin and fibroblast growth were obtained within three days of nerve processing. Characterization of the initial, expanded, and cryopreserved cell products confirmed maintenance of SC identity, viability and growth rates throughout the process. Most importantly, SCs retained their sensitivity to mitogens and potential for differentiation even after cryopreservation. To conclude, this easy-to-implement and clinically relevant protocol allows for the preparation of expandable homogeneous SC cultures while minimizing time, manipulation of the cells, and exposure to culture variables.

A vast literature on cultured Schwann cells (SCs) has been available since the mid-1970s, when it was discovered that SCs could be isolated from neurons and grown independently of the trophic support provided by their association with axons^1^. Several methods are currently available for the culturing of embryonic, postnatal, and adult SCs. Essentially, these methods differ in the type and age of the tissue used as starting material, the inclusion of a pre-degeneration step, and the purification system used to eliminate contaminating fibroblasts^2^,^3^. To date, most published protocols have relied on the use of postnatal sciatic nerve and embryonic dorsal root ganglion explants as sources of SCs, due to the advantage they provide for effective enzymatic dissociation and establishment of purified expandable cultures. Early postnatal nerves are not only essentially devoid of myelin^4^, but also exhibit immature connective tissue layers that both facilitate enzymatic dissociation and reduce the load of contaminating cells^5^,^6^. In addition, postnatal SCs exhibit a significantly higher proliferation rate than adult cells cultured under similar conditions^7^,^8^.

The culturing of adult nerve-derived SCs is much more labor intensive, as some hard-to-overcome technical hurdles during the steps of nerve processing and cell purification can limit the efficient isolation of viable SCs. Two important challenges faced when using adult nerves as a source of SCs include the difficulty in separating nerve cells from the myelin debris and the existence of fully developed endo-, peri- and epineurial sheaths enriched in connective tissue that hinder activity of proteolytic enzymes. Typically, the digestion of the tissue and removal of the myelin requires a prolonged incubation period with digestion enzymes, severe mechanical disintegration, and/or additional steps for myelin purification, which altogether compromises the recovery and viability of primary cell suspensions.

It has been shown that these hurdles can be overcome at least in part by introducing a step of *in vitro* or *in vivo* pre-degeneration of the nerve tissue prior to enzymatic treatment. This step, which is intended to allow Wallerian degeneration to take place while concomitantly allowing SC dedifferentiation, proliferation and myelin degradation, has been shown to increase both the viability and yields of SCs obtained from adult nerves^6^,^9^,^10^,^11^,^12^,^13^,^14^. It has also been argued that *in vitro* pre-degeneration of adherent nerve tissue explants promotes the outgrowth of fibroblasts and contributes to reduce fibroblast contamination in the initial populations^11^. However, the requirement of a pre-degeneration step not only delays release of the nerve cells but also exposes them to potentially deleterious conditions such as prolonged hypoxia.

The goal of this study was therefore to develop a culture method that would efficiently procure primary adult nerve-derived SC populations while skipping the pre-degeneration phase. Reported here is a step-by-step protocol for the immediate dissociation of adult rat sciatic nerve tissue that consists of a series of versatile and easy-to-implement steps during nerve processing, cell plating, myelin removal, and SC enrichment. This protocol allowed us to harvest highly viable and purified SC populations as early as 3 days post-digestion. These SCs could be used directly in experimentation, expanded in number if necessary, purified of contaminating cells by magnetic cell sorting, and/or cryopreserved for long-term use. We confirmed that the myelin-free SC populations that are derived through this method are highly proliferative and retain their native phenotype and potential for differentiation. We also showed that critical steps in this process could be validated using cultures of rodent postnatal nerves. Overall, our studies support the feasibility to obtain substantial numbers of adult nerve-derived, purified SCs with minimal manipulation. We anticipate that, with minor modifications, this method can be adapted for use with a variety of nerve sources, species and stages of differentiation.

## Results

### Immediate enzymatic dissociation of adult rat sciatic nerve: nerve tissue processing, enzymatic treatment, and cell plating

Methods to isolate SCs from adult nerves traditionally involve an *in vivo* or *in vitro* pre-degeneration step, based on the need to facilitate myelin clearance prior to enzymatic dissociation^11^. To avoid this typically lengthy phase, we herein implemented a series of measures to promptly dissociate the nerve fibers, remove the myelin debris, and recover highly viable populations of adherent cells.

**Figure 1:**
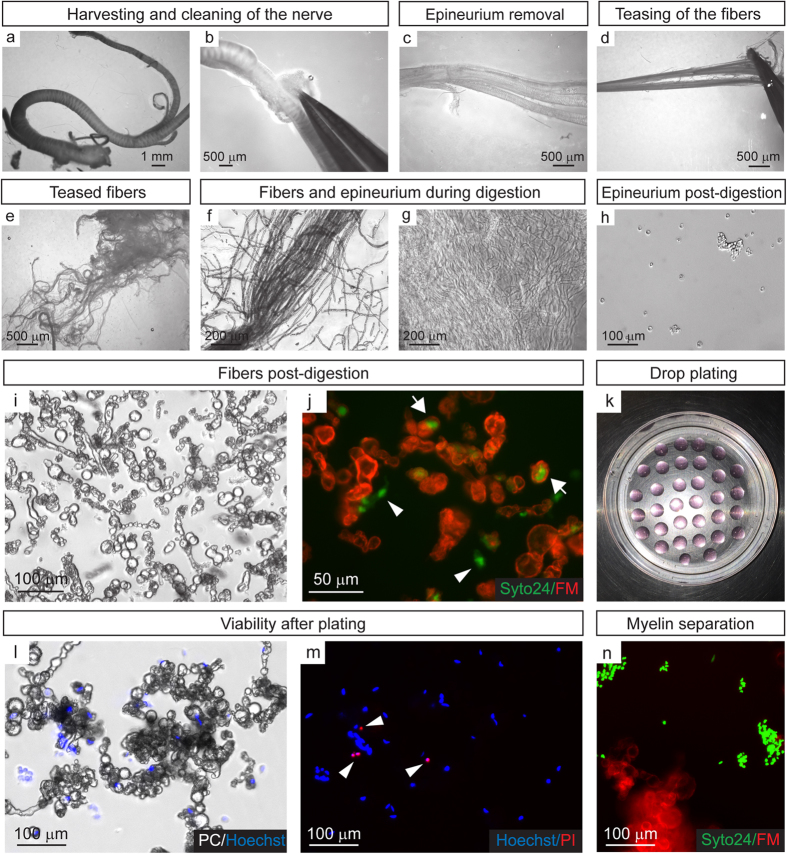
Immediate enzymatic dissociation of adult rat sciatic nerve tissue. The most significant steps during nerve processing and isolation of the cells from both the nerve fibers and epineurium are shown. (**a**) Intact sciatic nerve biopsy right after harvesting. (**b**) Removal of non-peripheral nerve tissue. (**c**) Dissection of the epineurium layer. (**d**) Separation of the nerve fascicles and teasing of the fibers with fine forceps. (**e**) Low magnification view of finely teased nerve fibers prior to enzymatic dissociation. (**f,g**) Higher magnification view of partially digested fibers (**f**) and epineurium (**g**), respectively, one hour after incubation in collagenase/dispase cocktail. (**h,i**) Preparations derived from digested epineurium (**h**) and fibers (**i**) immediately before plating. Low magnification phase contrast (PC) images of the end product of immediate enzymatic digestion are shown. (**j**) Higher resolution view of the product shown in (**i**) denoting the cellular versus the myelin fractions that result from enzymatic treatment. Fluorescence microscopy image showing cell nuclei labeled in green with Syto24 and myelin debris stained in red with Fluoromyelin (FM). Note that abundant myelin-free cell nuclei were apparent in these fractions (arrowheads) along with nuclei associated with myelin fragments (arrows). (**k**) Drop plating method onto PLL-laminin coated 10 cm dishes for enhanced cell attachment and myelin separation. Drops (30 μl each) contain cells from the product shown in (**j**) seeded at a density of ~30 drops/plate. (**l,m**) Phase contrast (PC) (**l**) and fluorescence (**m**) microscopy images showing a high percentage of viable cells 24 h after plating. Cells were labeled with Hoechst (blue, total nuclei) and propidium iodide (PI) (red, dead nuclei). Dead cells are indicated by arrowheads. (**n**) Fluorescence microscopy image revealing that myelin debris (Fluoromyelin Red) float and separate from adherent cells (Syto24).

The overall procedure is illustrated in Fig. 1, which depicts the most relevant steps required for nerve processing, enzymatic dissociation, and cell plating using the nerve material from one adult female Sprague Dawley rat. First, both sciatic nerves were harvested under aseptic conditions and stripped off their associated adipose and muscular tissue using fine forceps (Fig. 1a,b). Subsequently, the outermost connective tissue layer, the epineurium, was removed as one single sheath and collected for enzymatic digestion and cell composition analysis, as this layer is known to be the main source of contaminating fibroblasts in SC cultures^5^,^6^. The dissected epineurium was easily recognized due to its whitish color and elastic properties (Fig. 1c). In parallel, with the help of fine forceps, the fibers were extensively teased until no individual fascicles were evident (Fig. 1d). The teasing step was carried out until all fascicles were separated into individual fibers regardless of their initial caliber (Fig. 1e). The epineurium and teased fibers were subjected to digestion with an enzymatic cocktail composed of dispase II and type I collagenase^6^. Figure 1f,g displays representative images of partially digested tissue from the teased fibers and epineurium, respectively, to reveal the progression of the digestion and the purity of each preparation. Enzymatic treatment was typically performed overnight to maximize effectiveness of digestion and ensure cell release without the need for mechanical dissociation. Lastly, the end products of enzymatic digestion were filtered and subsequently collected by centrifugation.

Fluorescent staining with Syto24, a general nuclear marker, and Fluoromyelin Red (FM), a selective myelin stain, indicated that digestion of the teased nerve fibers rendered abundant cells that were found in isolation or in close association with small myelin fragments (Fig. 1i,j). In contrast, digestion of the epineurium rendered a clear and homogeneous cell preparation that consisted mainly of single cells that typically associated in small clumps prior to or after plating (Fig. 1h). For plating, the cells were collected in a small volume of serum-supplemented Dulbecco’s Modified Eagle Medium (DMEM) and plated as 30 μl-drops onto air-dried dishes coated sequentially with poly-L-lysine (PLL) and laminin. Plating was carried out in either 10 cm dishes or 24-well plates (Fig. 1k, shown only for 10 cm plates) for the purpose of cell expansion and fluorescence microscopy analysis, respectively. The drop plating method facilitated the recovery of viable adherent cells while preventing the attachment of myelin debris. This is illustrated in Fig. 1l–n, which provide representative pictures of cells within the drops as visualized by phase contrast (PC) (Fig. 1l) and fluorescent microscopy using a combination of Hoechst/propidium iodide (PI) (Fig. 1m) and Syto24/Fluoromyelin (Fig. 1n). Determination of cell viability immediately after plating rendered <10% cell death based on PI incorporation (Fig. 1m). In this and subsequent experiments, cell viability was determined by means of co-staining with PI, which selectively labels the nuclei of dead cells, and Hoechst, which, serving as a reference control, labels the nuclei of all cells.

To summarize, this method allowed the procurement of abundant primary cells from fibers and epineurium. Both the teasing of the fibers and the drop plating method onto a highly adhesive laminin substrate were essential for the recovery of viable cells. By opening the entire nerve into low diameter individual fibers, the teasing step greatly increased surface area and facilitated enzyme penetration into the tissue to ensure cell release. By suspending the cells in small drops, the plating method allowed high cell densities per unit surface, thus contributing to survival of the adherent populations. Of note, the drop plating method is adaptable for use in a variety of plate formats, including multi-well dishes.

### Recovery of highly viable SC populations with minimal myelin contamination: cell survival and phenotypic characterization of the acutely isolated preparations

**Figure 2:**
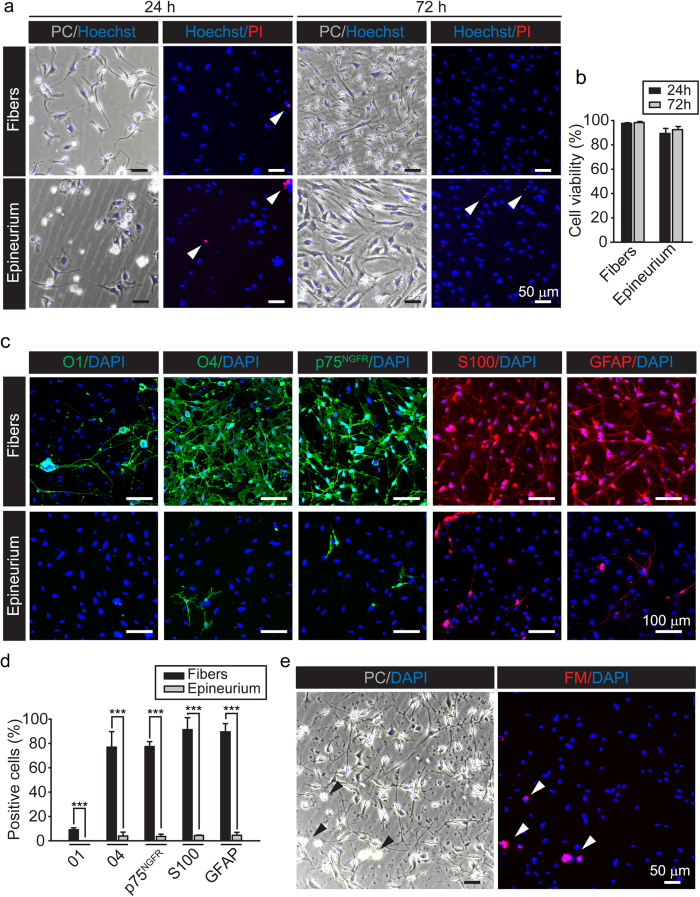
Acute isolation of highly viable adherent SCs with negligible myelin debris. (**a**,**b**) Viability assays based on Hoechst/PI incorporation in cultures derived from teased fibers and epineurium at 24 and 72 h after the initial plating in DMEM medium supplemented with 10% FBS, 10 nM neuregulin and 2 μM forskolin. Phase contrast (PC) and fluorescence microscopy images of representative areas of cultures from fibers (upper panels) and epineurium (lower panels) stained with a combination of Hoechst/PI are shown in (**a**). A grouped bar chart containing the quantification of the image data is shown in (**b**). Bars represent mean ± SD. Cell death was negligible in adherent cultures from both fibers and epineurium (arrowheads in **a**). In fibers, viability was estimated to be 97.5 ± 0.5% and 98.2 ± 1.0% at 24 and 72 h after plating, respectively. In epineurium, viability was 89.3 ± 4.0 and 92.4 ± 2.7%, respectively, when documented at the same time points. The health and potency of the cells was also manifested by the increase in the area covered by cells/cell processes as a function of time in culture. (**c,d**) Confirmation of SC phenotype and purity of the cultures isolated from fibers and epineurium. Low magnification (10x) images (**c**) and quantification (**d**) of cultures immunostained with antibodies against O1, O4, p75^NGFR^, S100, and GFAP. Immunostaining for all markers was performed in live cell cultures with the exception of S100 and GFAP. Quantitative results derived from image analysis are expressed as the percentage of cells exhibiting specific strong expression of each selected marker (Bars represent mean ± SD, ***p < 0.001). As expected, SCs were recovered in the fiber-derived populations. Note that myelin-associated O1 positive SCs were absent in epineurium cultures, consistent with the absence of myelin debris. (**e**) Myelin-specific staining of fiber-derived cultures. Fluoromyelin staining revealed only a few clumps of loosely attached myelin debris (shown by arrowheads) 72 h after plating.

To begin characterizing the cell populations obtained by immediate dissociation of nerve tissue, we examined the survival, growth and phenotype of the adherent cultures established from both the fibers and epineurium. A quantitative analysis of cell viability revealed a high percentage of live cells in both the fiber- and epineurium-derived cultures (Fig. 2a,b). To identify the phenotype of the isolated cells we performed an immunofluorescence microscopy analysis of various SC-specific markers in cultures from fibers and epineurium. As expected, cells expressing O4 (sulfatide), p75^NGFR^, S100, and GFAP (glial fibrillar acidic protein), which are widely used markers for peripheral glia^15^,^16^,^17^, were predominantly found in the populations derived from fibers (Fig. 2c,d). Some highly branched cells typically associated with myelin debris were identified based on their high levels of cell surface galactocerebroside (O1), a myelin lipid. These O1 positive cells were lost within a few days in culture, which is possibly due to their *in vitro* dedifferentiation to a less mature O1 negative phenotype (Fig. 2c,d).

Overlapping images of phase contrast microscopy and staining with Fluoromyelin Red further revealed that the cultures consisted predominantly of individual adherent cells, small adherent cell clumps and occasional myelin debris (Fig. 2a,e). The remaining floating myelin was easily removed by washing or changing medium within 2–3 days post-plating. Images provided in Figs 1l and 2a serve to compare the amount of myelin debris before and after washing, respectively. It is likely that the majority of the O1 positive, myelin-associated SCs were not efficiently procured from the onset, as these cells could fail to attach to the substrate or become easily lost during myelin removal (Fig. 2c).

In summary, these results indicate that provision of a highly adhesive substrate combined with plating of the cells in small individual drops favors a rapid attachment of the cells while minimizing myelin contamination and maintaining the health of the initial cell populations. An efficient procurement of clearly distinctive adherent phenotypes can be obtained from the dissociated fibers and the epineurium. The essentially myelin-free cultures derived from the teased fibers exhibit typical SC characteristics, which offers the potential for use in experimentation as early as 3 days post-plating.

### Recovery of highly proliferative SC populations with minimal fibroblast contamination: Characterization of the purity, growth and expansion potential of freshly isolated cells

One major setback in establishing and maintaining SC cultures from adult nerves is the rapid increase in the number of fibroblasts that typically overrides the number of SCs^18^. In order to maintain a low rate of fibroblast contamination and allow for a rapid expansion of the SC population, the culture medium was supplemented with a combination of neuregulin and forskolin as early as 1 day after plating. The expansion medium, herein referred to as mitogens medium, essentially consisted of DMEM containing 10% fetal bovine serum (FBS), 10 nM neuregulin and 2 μM forskolin. This medium was used throughout unless otherwise noted. The use of a neuregulin-based media formulation has been standard^6^ since it was discovered that the addition of a soluble neuregulin peptide mimics the action of axon-bound neuregulin, which is the natural mitogenic factor for developing SCs^19^. The addition of forskolin, a direct activator of the adenylyl cyclase and inducer of intracellular cAMP, is required to both synergistically enhance neuregulin-dependent SC proliferation and reduce fibroblast contamination, as S-phase progression in fibroblasts is attenuated by provision of cAMP-inducing agents^20^,^21^,^22^.

**Figure 3:**
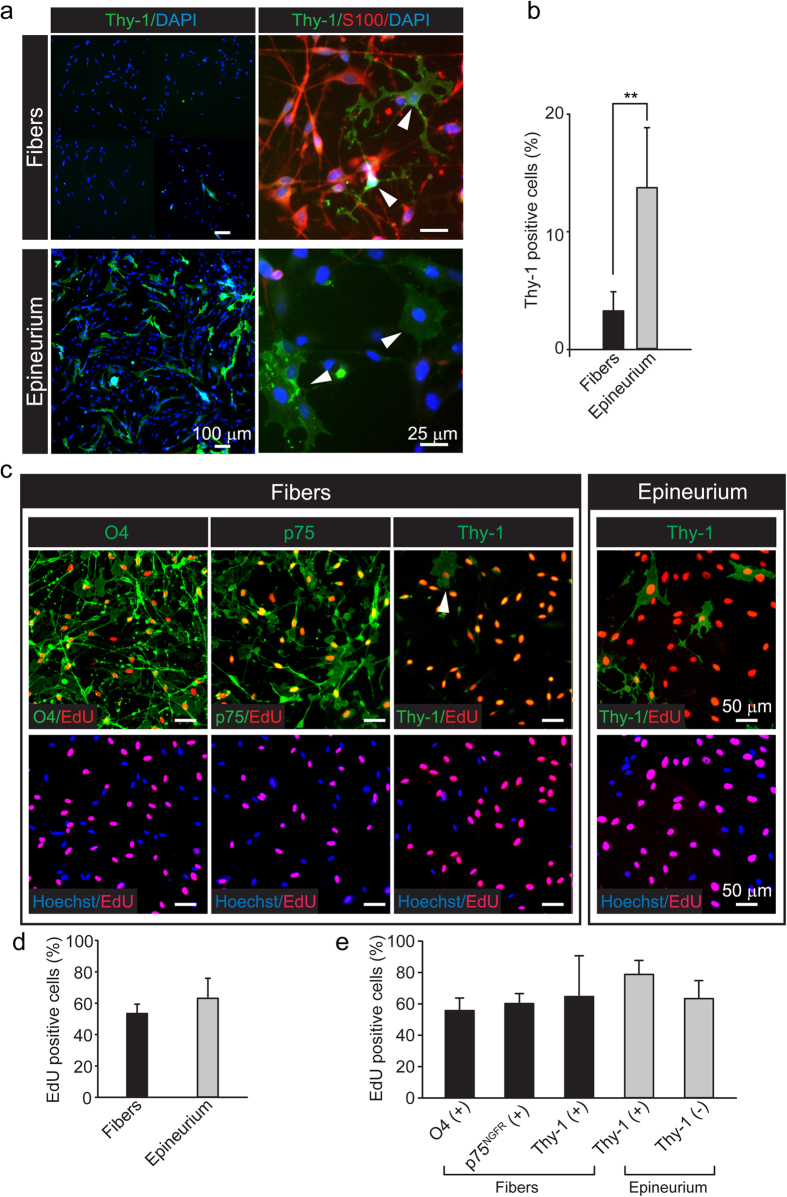
Proliferative capacity of the cell products from fibers and epineurium at 3 days post-plating. (**a**,**b**) Fluorescence microscopy (**a**) and quantitative analysis (**b**) of Thy-1 expression in fiber- and epineurium-derived cultures (bars represent mean ± SD, **p < 0.01). A panoramic, low magnification view of the cultures is shown to reveal the extent of fibroblast contamination (left images). Higher magnification images (right images) of cultures double immuno-stained with Thy-1 and S100 antibodies are shown to confirm the specificity of labeling and the morphology of the cells in each preparation. Fibroblasts are indicated by arrowheads. (**c,e**) Fluorescence microscopy analysis of EdU incorporation along with immunostaining with O4, p75^NGFR^ and Thy-1 antibodies. Representative images (**c**) and quantitative analysis (**d,e**) of cultures labelled with EdU (red) and the markers indicated in the figure (green) are provided. As shown by the graph displayed in panel (e), more than half of the isolated SCs were identified as actively proliferating cells (bars are shown as mean ± SD).

To assess the magnitude of fibroblast growth in the initial cell populations, the cultures were analyzed for purity by means of immunofluorescence microscopy analysis. In addition, the proliferative potential of SCs and fibroblasts was determined by means of EdU labeling, as incorporation of the thymidine analog EdU into nuclear DNA provides a reliable measure of S-phase entry in fixed cells. Purity determinations were done by live cell immunolabeling with antibodies against the fibroblast marker Thy-1 (CD90), a cell surface glycosylphosphatidylinositol-anchored glycoprotein expressed in fibroblasts but not SCs^23^,^24^. To detect Thy-1 in rat cultures, we used monoclonal antibodies against the murine allelic isoform Thy1.1. Results revealed that populations derived from the teased fibers contained a minimal percentage of contaminant Thy-1 positive fibroblasts, as only 3.28 ± 1.62% of all cells displayed immunoreactivity for cell surface Thy-1 (Fig. 3a upper panel and b). In fact, Thy-1 positive cells were mostly recovered in the epineurial fraction, along with a proportion of a still unidentified population of Thy-1 negative cells that also stained negative for S100, GFAP and other SC-specific markers (Fig. 3a, shown only for S100). Epineurial cells were notoriously different from fiber-derived cells, as judged by their enlarged nuclei and flattened morphology (Figs 2a and 3a).

Results from EdU incorporation assays indicated that the provision of chemical mitogens in the presence of FBS supported active proliferation in cells from both fibers and epineurium as ~60% of these cells incorporated the EdU label over a time period of 3 days (Fig. 3c,d). The percentage of proliferating, EdU positive cells was also calculated individually for each cell type population (Fig. 3e). These results confirmed that an important proportion (>55%) of the cells identified as SCs on the basis of O4 or p75^NGFR^ expression labeled positive for EdU incorporation (Fig. 3c,e). Thy-1 positive cells also proliferated actively (Fig. 3e) despite their low representation in the fiber-derived populations (Fig. 3b). The percentage of proliferating Thy-1 positive cells was determined to be 64.67 ± 25.94% and 78.69 ± 9.00% in the fiber and epineurium fractions, respectively (Fig. 3c,e). Of note, highly proliferative Thy-1 negative cells of still undetermined identity were typically found in the epineurium rather than the fibers (Fig. 3c, right panels, and 3e).

**Figure 4:**
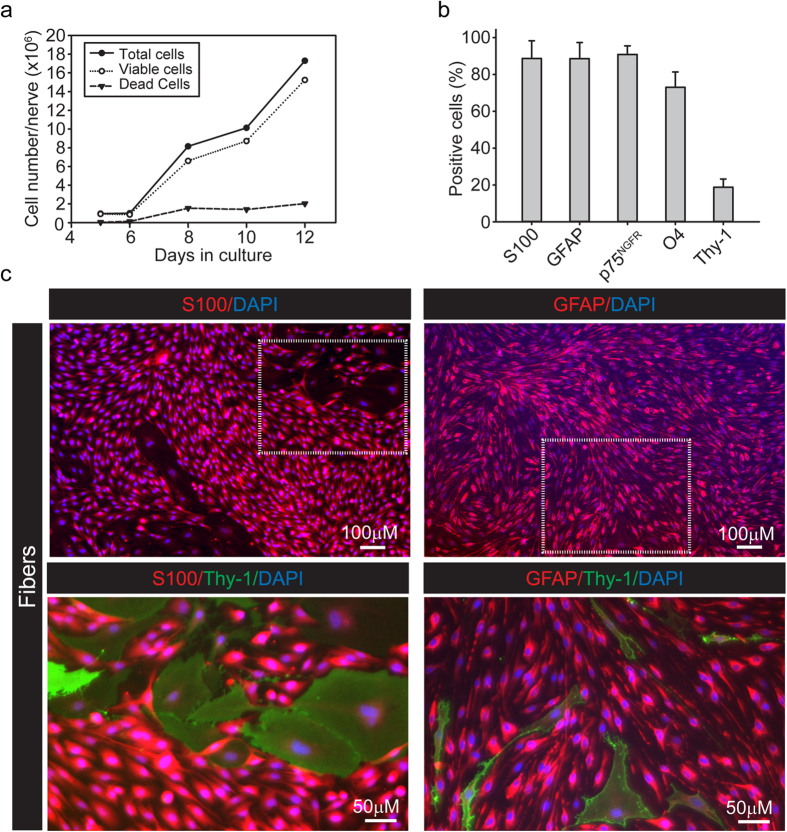
Characterization of the growth and phenotype of cultures derived from the nerve fibers. (**a**) Temporal progression of cell expansion and viability determinations by Trypan blue exclusion assays. The growth curve is representative of the progression of a selected culture initially established in a 10 cm dish, essentially as illustrated in Fig. 1k. Data was normalized to represent the number of total, viable and dead cells per nerve biopsy at the indicated time points. (**b,c**) Cell type composition of the expanded cultures at 12 days post-plating. Quantification (**b**) and representative images (**c**) of immunofluorescence microscopy imaging of cultures stained with S100, GFAP and Thy-1 antibodies (bars represent mean ± SD). Low magnification images of cultures growing in 24-well dishes are shown (**c**, top panel) to reveal the overall appearance of high density cultures consisting of a high proportion of GFAP positive, S100 positive SCs (red). In (**c**), bottom panels, selected areas (white dashed rectangles) are displayed at higher magnification to reveal the presence of contaminating Thy-1 positive fibroblasts. SCs could be easily recognized by their characteristic spindle-shaped morphology. Fibroblasts were heterogeneous in both shape and size and also exhibited variable levels of cell surface Thy-1 expression.

The expansion potential of the fiber-derived cultures in mitogen-containing medium was determined by measuring the temporal increase in cell number using cells collected in suspension after trypsinization. Quantification of cell number at time intervals of 1–2 days, as indicated in Fig. 4a, revealed that cells rapidly expanded in number and remained highly viable throughout a time period of 12 days after culture initiation. Data analysis of the growth curves determined that the cells duplicated in number every 2.2 days on average, which was consistent with previous reports^6^,^19^,^25^. Importantly, this data showed that total cell yields in the order of 8 × 10^6^ cells per nerve biopsy can be obtained as soon as 10 days after the initial plating. An immunofluorescence microscopy analysis of the expanded cultures confirmed that the majority of the cells exhibited a typical SC phenotype characterized by high levels of expression of S100 (88.7 ± 9.5%), GFAP (88.6 ± 8.8%), p75^NGFR^ (90.8 ± 4.7%), and O4 (73.0 ± 8.3%) (Fig. 4b,c). Expanded cells also exhibited the expected elongated, bipolar morphology and ability for cell-to-cell alignment and bundle formation with increased cell density. Nevertheless, expansion of the SC population concomitantly occurred with an increase in the proportion of contaminating fibroblasts (Fig. 4b,c). Notably, Thy-1 positive cells increased from 3.28 ± 1.62% (Fig. 3b) to 21.1 ± 4.4% in less than 10 days of culture (Fig. 4b,c).

Overall, these results underscored the high potential for amplification of the fiber-derived cultures as well as the maintenance of the SC characteristics after expansion. Nevertheless, they also revealed the rapid progression of fibroblastic growth despite the use of a SC-specific medium formulation.

### Fast and efficient separation of SCs from contaminating fibroblasts via magnetic cell sorting: purification based on Thy-1 or p75^NGFR^ cell surface immunolabeling

Purification of primary cell cultures by magnetic activated cell sorting (MACS) allows the rapid separation of different cell populations whilst maintaining their viability and biological activity^26^,^27^. Thus, we used Miltenyi’s MACS technology to achieve efficient separation of the two main populations occurring in the expanded cultures derived from the adult nerve fibers, i.e. SCs and Thy-1 positive fibroblasts. To confirm the effectiveness of purification by MACS, we performed parallel experiments using primary cultures derived from postnatal rodent nerves, as these cultures typically contain a proportion of contaminating Thy-1 positive fibroblasts (Supplementary Fig. S1a). For MACS, SCs and fibroblasts were discriminated and separated from one another on the basis of their strong and homogeneous levels of expression of cell surface p75^NGFR^ (positive selection) and Thy-1 (negative selection), respectively. Although O4 offers an alternative means for specific live cell immunolabeling of SCs in general, O4 expression is often heterogeneous in cultured SCs. Variable levels of O4 expression were occasionally noticed in cultures of adult SCs but most strikingly in cultures derived from postnatal nerves, where only a small proportion (~20%) of the SCs exhibited detectable levels of O4 on their surface (Supplementary Fig. S1a). Given the prospective variability in the levels of O4, and the fact that its expression is highly dependent on environmental factors such as cAMP^28^, we disregarded the use of O4 immunolabeling for positive SC selection by MACS. Instead, we used the more widely expressed membrane antigen p75^NGFR^ for both purification and characterization. The intracellular antigens S100 and GFAP were used along with p75^NGFR^ as reliable markers to confirm the purity and identity of the cultures prior to and after separation.

**Figure 5:**
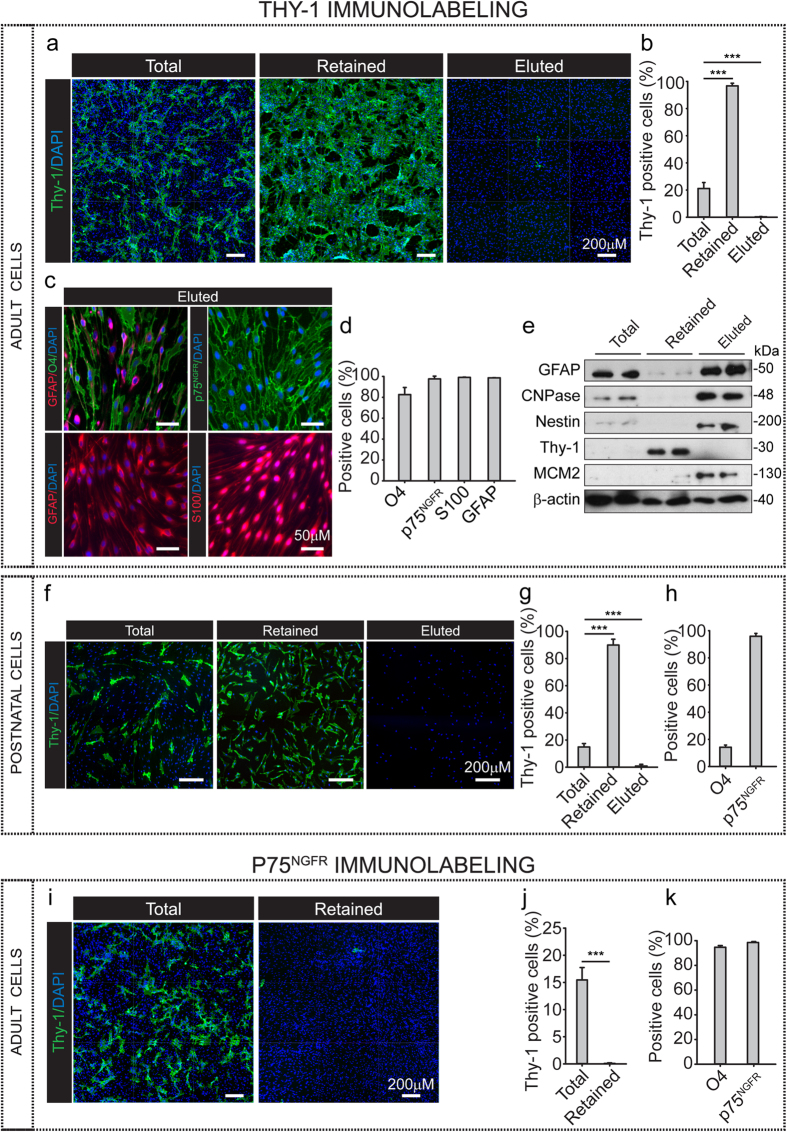
Separation of SCs and fibroblasts by MACS after cell surface immunolabeling with Thy-1 or p75^NGFR^ antibodies. (**a**–**h**) MACS separation of Thy-1-labeled cells from acutely isolated adult (**a**–**e**) and postnatal (**f**–**h**) rat nerve-derived cultures. Labeling and purification was performed as described in Methods. Cells were analyzed by immunofluorescence microscopy prior to (total fraction) and after MACS separation using the markers indicated in the figure (**a**,**c**,**d**,**f**,**h**). The retained and eluted fractions from each purification round were collected and analyzed in parallel. Low magnification images of cultures stained with Thy-1 (**a,f**) are shown along with a quantification of the results (**b,g**, bars represent mean ± SD, ***p < 0.001). Note the complete elimination of fibroblasts in preparations from both adult and postnatal sciatic nerves. An estimation of the percentage of remaining fibroblasts in the eluted fractions from adult and postnatal cells determined that only 0.3 ± 0.2% and 0.8 ± 1.0% of the cells, respectively, expressed Thy-1 immunoreactivity. Prior to purification, samples from adult and postnatal cells contained 21.11 ± 4.35% (**b**) and 14.83 ± 2.56% (**g**) of Thy-1 positive cells, respectively. In (**e**), duplicate samples from independent culture wells were analyzed by Western blot using antibodies against Thy-1, GFAP,CNPase, nestin and MCM2 (proliferation marker). Expression of β-actin served for reference control and confirmation of equal protein loading. (**i**–**k**) MACS separation of p75^NGFR^-labeled cells from acutely isolated adult nerve cultures (**a**–**e**). Immunofluorescence image analysis of the total and retained fractions confirmed the effectiveness of separation (**i**). A quantification analysis of the cells present in the retained fraction confirmed that the percentage of Thy-1 positive cells was as low as 0.1 ± 0.1% (**j**) and that the remaining of the cells (98.5 ± 0.8%) were identified as p75^NGFR^ positive SCs (**k**) (bars = mean ± SD, ***p < 0.001).

For cell purification, confluent cultures of adult and postnatal SCs growing in 10 cm dishes were enzymatically dissociated to obtain a single cell suspension. Immediately thereafter, the cells were subjected to sequential labeling with primary antibodies (Thy-1 or p75^NGFR^) and secondary antibodies conjugated with magnetic microbeads. Separation of labeled and non-labeled cells was carried out by passage through magnetic columns. Data shown in Fig. 5 depicts the results of representative MACS purification experiments based on Thy-1 cell immunolabeling using adult (Fig. 5a–e) and postnatal (Fig. 5f–h) nerve-derived preparations. Data shown in Fig. 5i–k depicts the results of a representative purification experiment based on p75^NGFR^ immunolabeling. Immunofluorescence and western blot analysis of the resultant cell suspensions indicated a high efficiency of separation of SCs and fibroblasts independently of the method used (i.e. positive or negative selection by MACS) and the age of the starting material (i.e. adult or postnatal cells). A quantitative analysis of the eluted and retained fractions from Thy-1 and p75^NGFR^ immunolabeling experiments, respectively, confirmed that the resultant SC preparations were essentially devoid of contaminating Thy-1 positive cells (Fig. 5b,g,j). Western blot analysis of the SC-specific markers GFAP, CNPase (2′,3′-cyclic-nucleotide 3′-phosphodiesterase) and nestin not only confirmed the purity of the fractions but also highlighted the enrichment of the SC population over that of Thy-1-expressing cells (Fig. 5e).

In addition, evidence indicated that the MACS protocol did not alter the identity or function of the purified cells regardless of the labeling method used. Firstly, we confirmed that the purified SC preparations derived from adult (Fig. 5c,d) and postnatal (Supplementary Fig. S1b,c) cultures contained viable cells that were essentially undistinguishable from the parent (non-purified) cells based on their morphology and the high levels of expression of the SC-specific markers p75^NGFR^, S100 and GFAP (Fig 5d,h,k, and Supplementary Fig. S1b,c). As expected, the purified SCs readily adhered to the laminin substrate by extending processes (Fig. 5c and Supplementary Fig. 1c) and responded to mitogenic stimulation by undergoing proliferation. The latter was inferred by the higher levels of expression of the G1-S marker minichromosome maintenance complex component 2 (MCM2) in the purified populations (Fig. 5e).

In sum, MACS technology offers a rapid and efficient means to specifically target and readily separate SCs and fibroblasts from adult and postnatal nerve-derived cultures. The results from our characterization studies confirmed that both SCs and fibroblasts were suitable for experimentation immediately after separation by MACS. Separation can be accomplished in minimal time (i.e. 2 hours or less) without compromising the health or biological activity of the purified cells.

### Effective cryopreservation of cell stocks allows for recovery of viable cells that retain their functional properties: functional assessment of proliferation and differentiation controls after freezing and thawing

Indefinite *in vitro* expansion of SC cultures is not generally recommended. This is due to the possible changes in cell behavior and associated phenotypic and genotypic alterations that may result from long-term exposure to proliferation medium^29^. Evidence has shown that SCs can be transformed and become growth factor-independent if culture is extended over 20 passages^30^,^31^. Even though producing fresh primary cultures would be ideal for experimental procedures such as transplantation, long-term maintenance of cultures is often laborious, time-consuming, or impractical for certain applications. As an alternative to the use of fresh tissues, cultures of primary cells have been cryopreserved to then be thawed and used at the moment of experimentation^31^,^32^.

**Figure 6:**
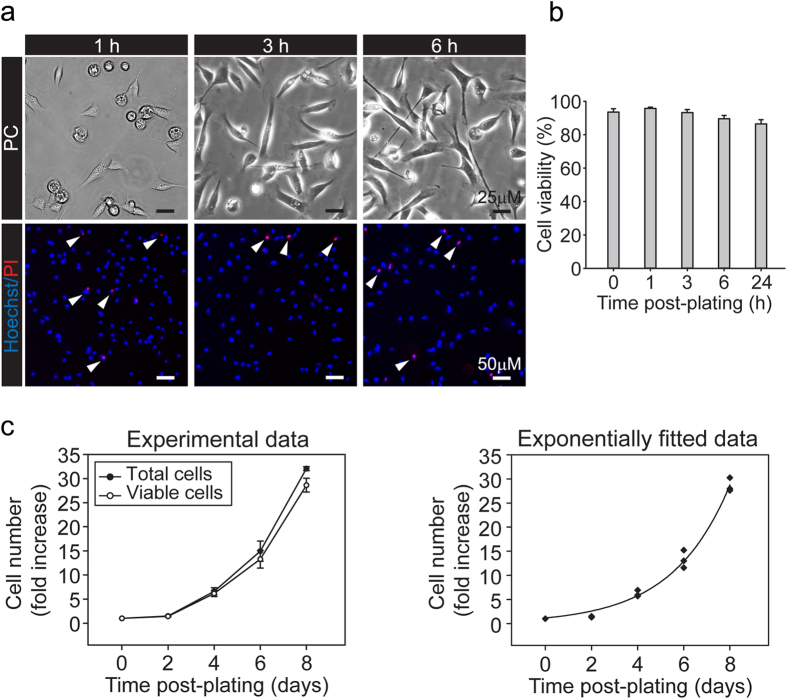
Analysis of viability and expansion potential of acutely isolated SCs obtained after cryopreservation. (**a,b**) Determination of viability and adhesion properties of cells obtained after freezing/thawing of cryogenic stocks. Acutely isolated adult rat SCs were plated in 10 cm dishes, maintained in culture for 12 days, purified by MACS (using Thy-1 antibodies) and cryopreserved 1 day after purification, as described in previous figures and the methods section. Subsequently, cells were thawed, re-plated onto PLL/laminin coated dishes in medium containing mitogens and analyzed by means of Hoechst/PI incorporation assays (**a**, lower panels) and phase contrast microscopy (**a**, upper panels) at the times indicated in the figure. Dead cells are indicated by arrowheads. A quantification of the percentage of live cells (PI negative cells) over time is provided in panel (b) (bars represent mean ± SD). (**c**) Determination of growth and expansion in medium containing mitogens. Cells shown in (**a**) were plated in 6-well plates at a density of 150,000 cells/well and maintained in mitogens medium for (**a**) total period of 8 days. Quantification of the number of total and viable cells at the indicated time points was performed by Trypan Blue exclusion assays. Data is expressed as the proportional increase in cell number (left panel) starting at the time of plating (t = 0). Data was also adjusted to an exponential curve (R^2^ = 0.9881) to calculate the duplication time (1.73 days) (right panel).

To test whether SC cultures from adult nerves could be subjected to cryogenic storage, we used a standard protocol for cell cryopreservation using a commercially available freezing medium. Figure 6 shows the results of cell recovery after thawing and re-plating of cryopreserved SC stocks onto dishes double coated with PLL and laminin. The attachment properties and the viability of the cells were determined by phase contrast and fluorescence microscopy after performing a vital staining with a combination of Hoechst and PI. As shown in Fig. 6a, our cryopreservation method allowed for the recovery of highly viable SCs that readily adhered to the substrate and extended processes as observed in preparations of fresh cells. Cell viability was estimated to be >85% over a time period of up to 24 h after plating (Fig. 6a,b). In addition, cryopreserved cells exhibited a high rate of growth in mitogen-containing medium (Fig. 6c). Data presented in the growth curve shown in Fig. 6c (left panel) was fitted to an exponential curve^33^ (Fig. 6c, right) that allowed us to estimate a population doubling time of 1.73 days. This value was consistent with the one calculated previously for exponentially growing populations of fresh cells (Fig. 4a).

**Figure 7:**
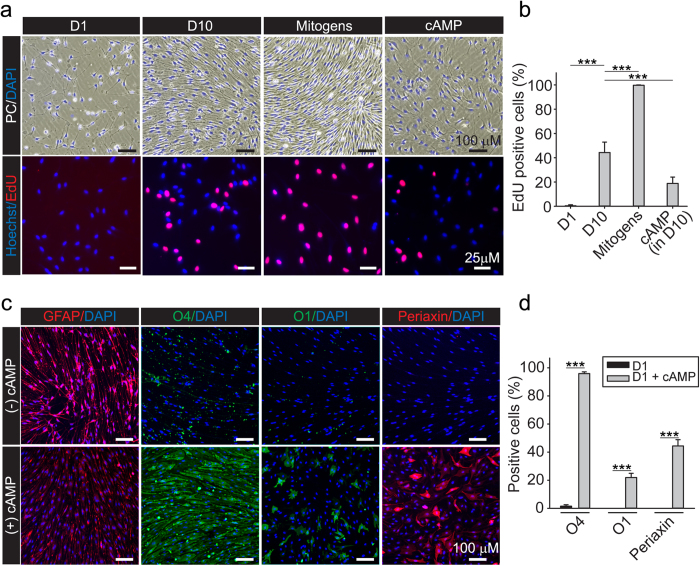
Analysis of proliferation and differentiation of isolated adult SCs obtained after cryopreservation. (**a**,**b**) Determination of the mitogenic responses to serum and soluble growth factors. Cells were plated as described in Fig. 6 in DMEM containing a non-mitogenic concentration (1%) of FBS (D1), 10% FBS (D10), 10% FBS supplemented with neuregulin and forskolin (Mitogens) or D10 in the presence of 250 μM CPT-cAMP (cAMP). Cells were monitored by phase contrast microscopy (upper panels) to reveal changes in cell morphology, alignment and density in each experimental condition. Treatment was carried out for 3 days. Results from EdU incorporation assays are shown in (**a**) (lower panels) and (**b**) (quantification of fluorescence microscopy data). (**c**,**d**) Determination of the differentiating responses to prolonged treatment with cell permeable cAMP analogs. Cells were plated as described in (**a**,**b**) with the exception of using D1 medium containing vehicle (control, −cAMP) or CPT-cAMP (250 μM) as inducer of differentiation (+cAMP). Cells were analyzed for the expression of the indicated markers by immunofluorescence microscopy 4 days after stimulation (bars = mean ± SD, ***p < 0.001).

We also confirmed that the rapidly expanding SCs derived from cryogenic stocks were able to retain their normal proliferation and differentiation controls. Results from phase contrast microscopy and EdU incorporation assays provided in Fig. 7a,b clearly indicated that the proliferation of the SC cultures was dependent on the presence of serum and the growth factors neuregulin and forskolin. Not only did SCs remain quiescent in the absence of growth factors (Fig. 7a, left panel), but they also stop proliferating upon the addition of high concentrations of cell permeable analogs of cAMP (Fig. 7a, right panel), a treatment that induces growth arrest and differentiation into a myelinating SC-like phenotype^34^. Prolonged treatment with cAMP analogs induced the expression of myelin-associated markers such as O1 and periaxin, and concomitantly reduced the expression of GFAP (Fig. 7c,d), as we reported previously using cultures of adult SCs obtained from pre-degenerated nerves^35^,^36^. As expected, cAMP treatment induced a morphological transformation of the cells along with high levels of expression of the cell surface antigen O4 (Fig. 7c,d). Altogether, these results confirmed the feasibility of including a cryopreservation step to store the excess of cells when necessary. Most importantly, cells obtained by immediate dissociation are expandable in the presence of mitogenic factors and retain their normal potential for differentiation and induction of myelin gene expression.

## Discussion

**Figure 8:**
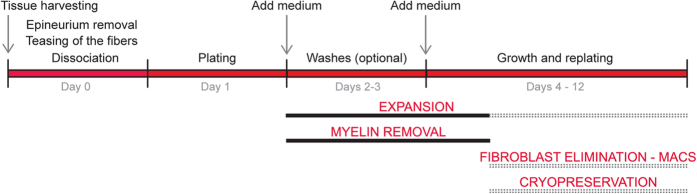
A rapid and versatile method for the isolation, expansion, purification and cryogenic storage of adult nerve-derived SCs. The schematic diagram summarizes the sequence of events in the method described in this study. The immediate dissociation of teased nerve fibers allows for an effective isolation of highly viable naïve SC populations that separate from myelin by differential adhesion to a laminin substrate. The naïve or expanded SCs, with or without purification, can be used for experimentation essentially at any step of the process. High cell yields in the order of 10^7^ cells/nerve can be obtained is less than 2 weeks of tissue harvesting.

This protocol implemented a series of new methodological steps for immediate cell isolation as a strategy to derive freshly isolated (parent) SC populations of increased homogeneity, purity and biological activity while minimizing time and exposure of the primary cells to potentially deleterious culture variables. This method is compatible with a variety of applications and offers the potential for clinical translation. As depicted in the schematic diagram presented in Fig. 8, our method consists essentially of the following basic steps: (1) Removal of the epineurium, extensive mechanical teasing of the nerve fibers, and enzymatic dissociation with a mixture of collagenase/dispase immediately after tissue harvesting (Day 0); (2) Plating the resultant cell suspensions as small droplets onto a highly adhesive PLL/laminin substrate to separate adherent SCs from floating myelin (Day 1); (3) Collection of the adherent cells for analysis or continued culture in medium containing chemical mitogens for the fast amplification of the SC population (Day 3); (4) Purification of the cultures by indirect magnetic cell sorting based on immunolabeling with p75^NGFR^ (positive selection) or Thy-1 (negative selection) antibodies, followed by direct analysis or continued culture; and (5) An optional cryo-preservation step for storage of the excess of cells.

It has been previously argued that enzymatic digestion of fresh intact tissue results in poor initial cell yields^10^,^11^,^37^. This argument has provided the basis for using a lengthy step of nerve tissue pre-degeneration prior to the enzymatic release of the cells. Pre-degeneration greatly facilitates myelin removal, as the products of axon degeneration induce active SC proliferation, dedifferentiation, and myelin clearance within the nerve explants, an advantage that can be further exploited for increased cell yield and survival after dissociation^6^. The time required for pre-degeneration ranges from 7 to 14 days^14^,^38^ and can be extended to up to 30 days depending on the protocol used^6^. A setback in this process is that an additional purification step for fibroblast removal may be required due to the fact that cultures typically become contaminated with hard-to-eliminate fibroblasts. Our method incorporated the following three key steps that are essential in order to successfully isolate myelin-free, pure adult SC cultures and eliminate the need for a pre-degeneration step. First, the removal of the epineurium as a single layer prior to enzymatic digestion eliminates the most prevalent source of fibroblasts and other potentially contaminating cells of high proliferative potential. Second, the extensive teasing of the nerve fibers allows for complete digestion of the tissue and the release of highly viable individual cells. Third, the drop plating method onto a laminin substrate allows rapid cell attachment and concomitant separation of myelin debris.

Even though the initial SC populations are nearly devoid of contaminating cells, prolonged *in vitro* culture inevitably leads to the concurrent expansion of highly proliferative fibroblast-like cells exhibiting a Thy-1 positive phenotype. The high growth rate of fibroblasts and their potential to overpopulate the glial cell populations has been one of the most common problems seen in adult SC culture^18^. Contaminant fibroblasts represent an important concern that should be minimized in the short-term, as they may induce a bias for interpretation of results of any particular study and increase scar tissue formation resulting in fibrosis if the intended use of the cells is transplantation^12^,^39^. We confirmed that the removal of epineurium layer was the first step needed to minimize fibroblast contamination. The use of forskolin-supplemented medium contributed to reduce the impact of fibroblast growth though it did not prevent the initial rapid expansion of the fibroblast population. In most published protocols fibroblast elimination has been achieved through various methods that exploit the differential biological properties of SCs and fibroblasts. These methods have used selective growth media^10^,^13^,^40^,^41^,^42^,^43^, anti-mitotic drugs^2^,^31^, differential adhesion to a plastic substrate^3^,^11^,^12^,^44^, and immunoselection^26^,^27^,^31^,^45^. Here, we have optimized two independent protocols for SC purification by either positive or negative MACS based on cell surface labeling with p75^NGFR^ or Thy-1 antibodies, respectively. High purity (>98%) of SCs over that of fibroblasts was achieved in less than 2 hours with the additional advantage to recover viable SCs and fibroblasts in independent fractions regardless of the protocol used. Each of these populations can in turn be used in experimentation, further expanded or cryopreserved without loss of viability or biological activity. Despite O4 immunolabeling provides valuable information for adult SC identification, it has limited use for MACS and purity determinations due to the intrinsic variability of O4 expression in populations of different origin and potential changing levels in response to factors present in the culture medium^28^. Of note, there is only one published protocol that has applied immediate adult nerve dissociation together with a selective growth medium containing D-valine for fibroblast depletion^42^. While this method eliminates the pre-degeneration step, it differs from ours in that a significant amount of time (19 days) is required to obtain SC cultures of enough purity. Moreover, our method offers the possibility to recover pure populations of fibroblasts, which may be prove beneficial for certain applications. It is becoming increasingly clear that peripheral nerve fibroblasts have a role in nerve regeneration by promoting neurite outgrowth^46^,^47^ and SC migration through secretion of growth factors such as neuregulin^46^,^48^ and the expression of extracellular matrix proteins^47^.

One important advantage of using an immediate dissociation protocol is that the impact of cell selection in culture and/or departure of the nerve cells from their original phenotype may be minimized. To address the maintenance of the SC phenotype in culture we performed a stepwise description of the prevalent phenotypes in the original and expanded populations. On the one hand, we characterized the expression of phenotypic markers and the proliferative potential of the freshly isolated endogenous SCs. On the other hand, we compared these naïve cells with those derived by short-term expansion in medium containing chemical mitogens both before and after subjecting them to purification and cryopreservation. Our studies revealed that both the initial and expanded SC populations were relatively homogeneous based on the expression of various SC-specific markers and that the cells retained basic SC-specific attributes such as the capacity to align with one another to form typical bundles of cells at confluency, a strict dependency on neuregulin for proliferation, and the ability to induce myelin-specific gene expression upon cAMP stimulation.

The collective methodological implementations were adjusted for use with adult rat sciatic nerve biopsies. Nevertheless, the method is flexible enough to be modified accordingly for use with other types of nerves, other species and stages of nerve development. We have already validated the reliability of the drop plating method, MACS purification and cryopreservation by using postnatal rat sciatic nerves. We also provide the first detailed description of a straightforward cryogenic storage method that guarantees recovery of highly viable, biologically competent cells. Cryopreservation has many advantages for delayed analysis, comparison of cells derived from independent batches, transfer and commercialization.

The adult SCs can suit the needs of wide variety of applications in studying their role in nervous system regeneration, demyelinating neuropathies, and uses in cell therapy for the treatment of spinal cord and peripheral nerve injuries^49^,^50^,^51^,^52^,^53^. In particular, the method used to separate the cellular versus the myelin components is simple, cost-effective, and clinically relevant. Our protocol is also compatible with methods and reagents already approved by the Food and Drug Administration (FDA) of the US for the manufacturing of autologous human SCs intended for use in the clinical treatment of spinal cord injury^52^. In envisioning a future translation of our methods to generate clinical grade human SCs we should point out that the conditions used for enzymatic digestion, plating, and growth in neuregulin- and forskolin-supplemented medium have previously been approved by the FDA for use in human subjects^52^,^54^. Clinically approved strategies for improving purity, such as selective adhesion of fibroblasts to plastic substrate, can be implemented when necessary. Despite MACS technology has not been used previously in the clinical production of SCs, there is at least one precedent for its use in clinically grade cellular products^55^. While most of the available methods for SC culture have focused on purity, viability and yields through optimization of the pre-degeneration step, these methods have disregarded factors such as the time needed to procure large enough cell populations at passage zero and the potentially adverse impact of this phase on the quality and biological activity of the cells. Some applications that use adult nerve-derived SCs, such as transplantation strategies in the spinal cord, may greatly benefit from the possibility of accelerating the isolation and purification of SCs so as to match the engrafting of autologous cells with the time when optimal axonal regeneration can be achieved.

To conclude, our protocol is, to our knowledge, the first optimized method that allows the rapid isolation of naïve SC populations that can be rapidly expanded, purified, or cryopreserved essentially at any stage without a loss in biological activity. Our method is suitable in diverse applications, such as cell therapy or drug discovery, and can be scaled up or down as needed in a variety of culture formats. A main advantage is the procurement of pure and homogeneous SC populations with as low as possible *in vitro* manipulation. Future directions include adaptation and optimization of this protocol for use with adult nerves from humans and other species used as models for human diseases.

## Methods

### Cultures of adult and postnatal rat Schwann cells

#### Tissue harvesting, removal of the epineurium and teasing of the fibers

The study protocol was approved by the University of Miami Animal Care and Use Committee. Experiments were conducted in accordance with the regulations and guidelines of The University of Miami Animal Care and Use Committee (IACUC), which supervises the work and acts in compliance with all USDA and NIH regulations governing the use of vertebrate animals for experimentation. Adult and neonatal sciatic nerves were obtained from adult female (3-month-old) and postnatal day-2 (P2) Sprague-Dawley rats, respectively. Sciatic nerves were dissected out immediately under aseptic conditions and placed in ice-cold Leibovitz’s L-15 Medium (L-15) medium containing gentamicin.

Each adult sciatic nerve was cleaned off excess tissue including muscle, fat and blood vessels while working under a Stemi 305 Stereo dissecting microscope (Carl Zeiss Microscopy GmbH, Germany) placed inside a horizontal laminar flow hood. After the removal of non-peripheral nerve tissue, each nerve biopsy was weighed and measured. Adult nerve fragments (~2.5 cm in length) weighed 33.9 ± 5.2 mg on average after cleaning. Subsequently, starting at the most proximal end to the spinal cord and with the help of fine forceps, the epineurium was mechanically removed as one single sheath. The epineurium and nerve fibers were individually collected and placed into fresh L-15-containing dishes. The nerve fascicles were extensively teased to the level of individual fibers using Dumont N° 4 and N° 5 fine forceps (Biology tips) with visualization through a dissecting microscope.

The teasing step consisted of repeated pulling of the individual nerve fascicles until fibers of the least possible caliber were obtained. On average, dissection and teasing of one nerve biopsy was completed within 30–45 minutes, depending on the operator’s technical skills. Tissues were transferred regularly to new dishes containing ice-cold L-15 medium for increased safety throughout the process of cleaning, removal of the epineurium and teasing of the fibers. Images of all these steps were acquired using an AxioCam ERc 5s camera coupled to the dissecting microscope and ZEN 2 lite Imaging software 2.0 (Carl Zeizz Microscopy GmbH).

Postnatal nerve fragments were cleaned off non-peripheral nerve tissues essentially as described above with the exception that the tissue was cut into small fragments using fine scissors, rinsed in cold L-15, and immediately subjected to enzymatic dissociation.

#### Enzymatic dissociation and initial plating

The teased adult sciatic nerve fibers, along with the respective epineurium layers collected in individual 60 mm plates, were subjected to enzymatic dissociation using a mixture of 0.25% Dispase II (Roche Diagnostics) and 0.05% type I collagenase (Worthington Biochemical Corp.) prepared in high-glucose DMEM (Thermo-Fisher). Tissue digestion was allowed to proceed overnight (average 15–18 h) at 37 °C in a 9% CO_2_ incubator. Digestion was regularly monitored under the phase contrast microscope following the addition of the enzymatic cocktail to confirm the progression of tissue digestion. Postnatal rat sciatic nerve fragments were dissociated by sequential treatment with 0.1% type I collagenase and 0.25% trypsin TRL3 (Worthington Biochemical Corp.). Incubation with each digestive enzyme was performed for 30 minutes at 37 °C inside a CO_2_ incubator. Enzymatic treatment was stopped in all cases by the addition of 40% FBS in Hanks’ Balanced Salt Solution (HBSS) followed by low speed centrifugation (188 × g, 10 min, 4 °C). The resultant cell pellets were resuspended in DMEM containing 10% FBS (GE Healthcare). Next, resuspended cells were gently passed through a small-bore fire polished borosilicate glass pipet, collected by low speed centrifugation and resuspended in DMEM-10% FBS containing antibiotics. Cells were plated as regularly dispersed drops (30 μl each) onto air-dried PLL-laminin-coated 10-cm dishes (for expansion) or 24-well plates (for image analysis). Cells were incubated overnight at 37 °C in a CO_2_ incubator without disturbing the droplets. Plastic cell culture dishes were coated by sequential incubation with 0.02% PLL solution prepared in distilled water (1 h at room temperature, RT) and 1 μg/cm^2^ of mouse laminin solution (Sigma) prepared in Dulbecco’s phosphate-buffered saline (DPBS) (overnight at 4 °C).

#### Characterization of the initial cell populations and determination of viability

The temporal progression of the digestion of the fibers and epineurium and the viability of the plated cells was determined by a combination of phase contrast and fluorescence microscopy analysis. Cell viability assays were performed immediately after dissociation and at different time points after plating. Dead cells were discriminated from total cells by staining with a combination of 1 μg/ml PI and 1 μg/ml Hoechst 33342, respectively. Alternatively, the fluorescent green dye Syto24 (Thermo-Fisher) was used at 1 μM to label total cell nuclei in samples containing abundant myelin debris. Fluoromyelin red (Thermo-Fisher) was used to label myelin in suspension and in cultures of adherent cells.

#### Cell expansion, cell counting and preparation of growth curves

Medium composed of DMEM- supplemented with 10% FBS, 10 nM neuregulin (recombinant heregulin-β1_177–244_ from PreproTech) and 2 μM forskolin (Sigma) was used for the growth and expansion of both adult and postnatal SCs unless otherwise noted. Medium was typically replaced every 2–3 days. Washes with L-15 or DMEM prior to feeding were carried out when abundant myelin debris was observed in suspension. Determination of cell number and viability was carried out to assess the temporal course of cell expansion. Briefly, cells were collected from their respective dishes by mild trypsinization. Trypan blue exclusion assay was then performed to discriminate live versus dead cells. Estimation of the total number of live/dead cells was done using a Bio-Rad TC20™ automated cell counter (Bio-Rad, Hercules, CA), according to the manufacturer’s instructions. In turn, quantitative data was used to construct growth curves and calculate the doubling time using the algorithm provided by http://www.doubling-time.com/compute.php^33^.

### Purification of SC cultures by magnetic cell separation

Two alternative, yet equally efficient methods for SC purification were developed. These methods were based on the selective expression of cell surface markers in SCs (p75^NGFR^) and fibroblasts (Thy-1), respectively. Fibroblast depletion was carried out through Thy-1 cell labeling via a protocol for negative selection whereas SC enrichment was carried out through the positive selection of p75^NGFR^-expressing cells. Indirect magnetic cell labeling and separation were performed essentially as recommended by the manufacturer (Miltenyi Biotec GmbH, Germany). Cells maintained in culture for 7–12 days after the initial plating (10 cm dishes) were used for purification using MACS.

#### Indirect magnetic labeling with Thy-1 and p75^NGFR^ antibodies

Briefly, the cells were detached from their PLL-laminin plates using 0.05% trypsin-EDTA (Thermo-Fisher), counted, and collected by centrifugation (188 × g, 8 min at 4 °C) prior to being labeled with Thy-1 or p75^NGFR^ antibodies. Typically, 5 × 10^6^ cells were labeled by resuspending them in 5 ml of Thy-1 or p75^NGFR^ hybridoma culture supernatant. Cell surface labeling was carried out for 20 minutes at 4 °C. The cells were collected by centrifugation and rinsed twice with buffer consisting of 0.5% bovine serum albumin (BSA) and 2 mM EDTA in phosphate-buffered saline (PBS) (pH 7.2), using 1–2 ml of buffer per 10^7^ cells. Subsequently, the cells were incubated for 15 minutes at 4 °C in 80 μl of BSA-EDTA-PBS buffer containing 20 μl of magnetic microbead-conjugated IgM or IgG anti-mouse antibodies (Miltenyi Biotec, GmbH) for Thy-1 and p75^NGFR^ labeled cells, respectively. Cells were washed, collected by centrifugation, and resuspended in BSA-EDTA-PBS buffer, according to the manufacturer’s recommendations.

#### Magnetic separation and plating

The magnetically labeled cell fractions were separated from unlabeled cells using pre-equilibrated MS columns (Miltenyi Biotec) placed in the magnetic field of a MACS Separator (Miltenyi Biotec). Each cell suspension was applied onto the column to separate the unlabeled cells (eluted fraction) from the labeled cells (retained fraction). Both the eluted and the retained cell fractions were counted and subsequently plated onto PLL-laminin-coated dishes for analysis of purity and identity. A sample of unpurified, non-labeled cells was used as a reference control for the identification of the total initial population.

### Cryopreservation of cell stocks

Purified or non-purified SCs were trypsinized, resuspended in DMEM-10% FBS, counted, and collected via centrifugation. A single cell suspension containing 1–2 × 10^6^ cells was resuspended in 1–1.5 ml of cold Recovery™ Cell Culture Freezing Medium (Thermo-Fisher), according to the manufacturer’s instructions. Cryogenic vials containing cell suspensions were placed inside a Mr Frosty^TM^ Freezing Container (Thermo-Fisher) and immediately transferred to −80 °C for 24 hours before being moved to liquid nitrogen for long-term storage. The viability of cell stocks after thawing using this method was typically ≥90% as determined by Trypan blue exclusion assays.

### Hybridoma cell culture

Conditioned culture supernatant produced *in house* from Thy-1, p75^NGFR^, O4 and O1 hybridoma cells was used as a source of monoclonal antibodies for all cell labeling and purification experiments. Hybridoma cell lines were grown in Iscove’s Modified Dulbecco’s Medium (IMDM, Thermo-Fisher), containing 10% FBS, 1% GlutaMAX^TM^ Supplement, 1% penicillin/streptomycin and 0.1% gentamycin.

### Proliferation and differentiation assays

Proliferation of cultured adherent cells was detected by means of the Click-iT® Plus EdU Alexa Fluor® 594 Imaging Kit (Thermo-Fisher)^56^. The ratio of proliferating (Edu positive) to total (Hoechst positive) cells was determined by fluorescence microscopy analysis.

Differentiation of isolated SCs was determined after treatment with phosphodiesterase-resistant cell permeable analogs of cAMP (Adenosine 3′,5′-cyclic monophosphate, 8-(4-Chlorophenylthio or CPT-cAMP, from EMD Millipore). For proliferation, cells were exposed for 3 days to the following conditions: DMEM-1% FBS (non-mitogenic medium used as a control for basal levels of proliferation), DMEM-10% FBS (medium that supports SC survival and moderately induces proliferation), DMEM-10% FBS containing 10 nM neuregulin, and 2 μM forskolin (medium that renders maximal levels of SC proliferation) and DMEM-10% FBS containing 250 μM CPT-cAMP (medium that induces cell cycle arrest and promotes SC differentiation). For differentiation, cells were incubated in the absence (control) or presence of CPT-cAMP (250 μM) in DMEM-1% FBS for 3–5 days. Cells were analyzed for morphological changes by phase contrast microscopy, proliferation (EdU incorporation), and expression of differentiation markers by immunofluorescence microscopy.

### Immunofluorescence microscopy

For live cell labeling of adherent cells, cultures were incubated with hybridoma conditioned medium (20 min, RT), rinsed three times with DPBS, and fixed with 4% paraformaldehyde (PF) prior to incubation with Alexa-conjugated secondary antibodies. For immunofluorescence microscopy of fixed cells, cultures were treated with 4% PF (20 min, RT) followed by treatment with −20 °C methanol (10 min). After three wash steps and blocking in 5% normal goat serum, cultures were incubated overnight at 4 °C with the appropriate dilution of the primary antibody (or conditioned medium), washed, and labeled with secondary antibodies as above. DAPI (4′-6-diamino-2-phenylindole, dihydrochloride, Thermo Fisher) or Hoechst 33342 (Sigma) at a dilution of 1 μg/ml in PBS were used as nuclear stains.

Conventional fluorescence microscopy image analysis was performed using an Olympus IX70 inverted fluorescence microscope equipped with a cooled digital CCD camera (SensiCam QE, Cooke Corp.). Panoramic views of cell cultures were obtained via automated fluorescence microscopy using a High Content Screening System (Cellomics ArrayScan VTI HCS Reader, Thermo-Scientific) equipped with a Hamamatsu ORCA®-ER cooled 12 bit grayscale digital CCD Camera. All images were digitally processed and arranged for presentation using Adobe Photoshop CS6 and Adobe Illustrator CS6. For cell quantification analysis, pictures from random fields in independent wells were taken at low magnification using 4X or 10X magnification objectives, and the number of cells labeled positive for each marker was determined in reference to the total number of cells based on DAPI or Hoechst staining. O1, O4, p75^NGFR^ and Thy-1 positive cells were classified as such when evident homogeneous membrane staining was appreciated. For proliferation and survival assays, the percentage of red stained nuclei with EdU or PI, respectively, was calculated in reference to the total number of cells counterstained with Hoechst.

### Western-blots

Protein samples were prepared by resuspending cells growing in 24-well plates in lysis buffer (50 mM Tris,150 mM NaCl, 1% SDS, 0.5 mM dithiothreitol) containing protease and phosphatase inhibitors. Protein concentration was quantified using the Pierce™ 660nm Protein Assay Reagent (Thermo Fisher Scientific). Samples were combined with SDS buffer (400 mM Tris/HCl, pH 6.8, 10% SDS, 50% glycerol, 500 mM dithiothreitol, 2 μg/ml bromphenol blue) and denatured by boiling. Equal protein samples containing 5 μg total protein were loaded and resolved in 10% polyacrylamide SDS gels. Fractionated proteins were transferred to PVDF membranes using Trans-Blot® Turbo^TM^ Transfer System (Bio-Rad). Membranes were blocked with 2% Amersham ECL Prime Blocking Reagent (GE Healthcare) in Tris-Buffer saline containing 0.05% Tween-20 and incubated overnight with each primary antibody (1:1,000). Horseradish peroxidase-conjugated antibodies, 1:5,000 (Santa Cruz) were used as secondary antibodies to detect immunoreactive protein bands by enhanced chemiluminescence using ECL Prime Western Blotting Detection Reagent (GE HealthCare) and Amersham Hyperfilm ECL (GE HealthCare).

### Antibodies

Anti-CNPase (catalog number C5922), was from Sigma (St. Louis, MO). Mouse anti-Nestin (catalog number MAB353) was from EMD Millipore (Darmstadt, Germany). Polyclonal rabbit S100 (catalog number Z0311) and GFAP (catalog number Z0334) antibodies were from DAKO (Carpinteria, CA). β-actin rabbit monoclonal antibody (catalog number 8457S) was from Cell Signaling Technology (Beverly, MA). MCM2 rabbit polyclonal antibody (catalog number A300-191A) was from Bethyl Laboratories. Anti-mouse IgM (catalog number 130-047-301) and IgG MicroBeads (catalog number 130-048-401) were from Miltenyi Biotec GmbH (Bergisch Gladbach, Germany). The O1 and O4 hybridoma cell lines were kindly provided by Dr. M. Schachner (Rutgers, Piscataway, NJ). Periaxin antibodies were a kind donation of Dr. Peter Brophy (University of Edinburgh, UK). The Thy-1 hybridoma cell line T11D7e2 (TIB-103™), and the p75^NGFR^ (clone 192) were from American Type Culture Collection (ATCC, Manassas, VA, USA).

### Statistical analysis

Statistical analysis was performed with SigmaPlot 12.0 software. Experimental data are expressed as the mean ± standard deviation (SD) of a representative experiment out of at least 3 independent experiments performed, unless otherwise noted. Statistical significance of the different conditions was evaluated by the Student t-Test or one-way ANOVA. For multiple comparisons, Bonferroni post-test was used. P values < 0.05 were considered statistically significant. Values of all significant correlations (p < 0.05) are given with their degree of significance (*p < 0.05, **p < 0.01 and **p < 0.001).

## Additional Information

**How to cite this article**: Andersen, N. D. *et al*. A rapid and versatile method for the isolation, purification and cryogenic storage of Schwann cells from adult rodent nerves. *Sci. Rep.* **6**, 31781; doi: 10.1038/srep31781 (2016).

## Supplementary Material

Supplementary Information

## References

[b1] Wood P. M. Separation of functional Schwann cells and neurons from normal peripheral nerve tissue. Brain Res 115, 361–375 (1976). 135599 10.1016/0006-8993(76)90355-3

[b2] Wei Y. . An improved method for isolating Schwann cells from postnatal rat sciatic nerves. Cell and tissue research 337, 361–369, doi: 10.1007/s00441-009-0836-4 (2009). 19639342

[b3] Haastert-Talini K. Culture and proliferation of highly purified adult Schwann cells from rat, dog, and man. Methods in molecular biology (Clifton, N.J.) 846, 189–200, doi: 10.1007/978-1-61779-536-7_17 (2012). 22367812

[b4] Kaplan S., Odaci E., Unal B., Sahin B. & Fornaro M. Chapter 2: Development of the peripheral nerve. International review of neurobiology 87, 9–26, doi: 10.1016/s0074-7742(09)87002-5 (2009). 19682631

[b5] Bunge M. B., Wood P. M., Tynan L. B., Bates M. L. & Sanes J. R. Perineurium originates from fibroblasts: demonstration *in vitro* with a retroviral marker. Science (New York, N.Y.) 243, 229–231 (1989). 10.1126/science.2492115 2492115

[b6] Morrissey T. K., Kleitman N. & Bunge R. P. Isolation and functional characterization of Schwann cells derived from adult peripheral nerve. The Journal of neuroscience: the official journal of the Society for Neuroscience 11, 2433–2442 (1991). 1869923 10.1523/JNEUROSCI.11-08-02433.1991 PMC6575499

[b7] Stewart H. J., Morgan L., Jessen K. R. & Mirsky R. Changes in DNA synthesis rate in the Schwann cell lineage *in vivo* are correlated with the precursor–Schwann cell transition and myelination. The European journal of neuroscience 5, 1136–1144 (1993). 7506619 10.1111/j.1460-9568.1993.tb00968.x

[b8] Woodhoo A. . Notch controls embryonic Schwann cell differentiation, postnatal myelination and adult plasticity. Nat Neurosci 12, 839–847 (2009). 19525946 10.1038/nn.2323 PMC2782951

[b9] Casella G. T., Bunge R. P. & Wood P. M. Improved method for harvesting human Schwann cells from mature peripheral nerve and expansion *in vitro*. Glia 17, 327–338, doi: 10.1002/(SICI)1098-1136(199608)17:4;327::AID-GLIA7;3.0.CO;2-W (1996). 8856329

[b10] Verdu E., Rodriguez F. J., Gudino-Cabrera G., Nieto-Sampedro M. & Navarro X. Expansion of adult Schwann cells from mouse predegenerated peripheral nerves. Journal of neuroscience methods 99, 111–117 (2000). 10936650 10.1016/s0165-0270(00)00221-1

[b11] Mauritz C., Grothe C. & Haastert K. Comparative study of cell culture and purification methods to obtain highly enriched cultures of proliferating adult rat Schwann cells. Journal of neuroscience research 77, 453–461, doi: 10.1002/jnr.20166 (2004). 15248300

[b12] Haastert K., Mauritz C., Chaturvedi S. & Grothe C. Human and rat adult Schwann cell cultures: fast and efficient enrichment and highly effective non-viral transfection protocol. Nature protocols 2, 99–104, doi: 10.1038/nprot.2006.486 (2007). 17401343

[b13] Pietrucha-Dutczakv M., Marcol W., Francuz T., Golka D. & Lewin-Kowalik J. A new protocol for cultivation of predegenerated adult rat Schwann cells. Cell and tissue banking 15, 403–411, doi: 10.1007/s10561-013-9405-x (2014). 24197905

[b14] Niapour N. . Efficacy of optimized *in vitro* predegeneration period on the cell count and purity of canine Schwann cell cultures. Iranian journal of basic medical sciences 18, 307–311 (2015). 25945245 PMC4414998

[b15] Jessen K. R. & Mirsky R. The origin and development of glial cells in peripheral nerves. Nat Rev Neurosci 6, 671–682 (2005). 16136171 10.1038/nrn1746

[b16] Buchstaller J. . Efficient isolation and gene expression profiling of small numbers of neural crest stem cells and developing Schwann cells. The Journal of neuroscience: the official journal of the Society for Neuroscience 24, 2357–2365, doi: 10.1523/jneurosci.4083-03.2004 (2004). 15014110 PMC6729482

[b17] Jacob C. Transcriptional control of neural crest specification into peripheral glia. Glia , doi: 10.1002/glia.22816 (2015). 25752517

[b18] Brockes J. P., Fields K. L. & Raff M. C. Studies on cultured rat Schwann cells. I. Establishment of purified populations from cultures of peripheral nerve. Brain Res 165, 105–118 (1979). 371755 10.1016/0006-8993(79)90048-9

[b19] Porter S., Clark M. B., Glaser L. & Bunge R. P. Schwann cells stimulated to proliferate in the absence of neurons retain full functional capability. The Journal of neuroscience: the official journal of the Society for Neuroscience 6, 3070–3078 (1986). 3760949 10.1523/JNEUROSCI.06-10-03070.1986 PMC6568804

[b20] Rahmatullah M. . Synergistic regulation of Schwann cell proliferation by heregulin and forskolin. Mol Cell Biol 18, 6245–6252 (1998). 9774641 10.1128/mcb.18.11.6245 PMC109211

[b21] Fregien N. L., White L. A., Bunge M. B. & Wood P. M. Forskolin increases neuregulin receptors in human Schwann cells without increasing receptor mRNA. Glia 49, 24–35, doi: 10.1002/glia.20091 (2005). 15390106

[b22] Heldin N. E., Paulsson Y., Forsberg K., Heldin C. H. & Westermark B. Induction of cyclic AMP synthesis by forskolin is followed by a reduction in the expression of c-myc messenger RNA and inhibition of 3H-thymidine incorporation in human fibroblasts. Journal of cellular physiology 138, 17–23, doi: 10.1002/jcp.1041380104 (1989). 2536035

[b23] Fields K. L. & Raine C. S. Ultrastructure and immunocytochemistry of rat Schwann cells and fibroblasts *in vitro*. Journal of neuroimmunology 2, 155–166 (1982). 6121824 10.1016/0165-5728(82)90006-6

[b24] Morris R. J. & Beech J. N. Differential expression of Thy-1 on the various components of connective tissue of rat nerve during postnatal development. Developmental biology 102, 32–42 (1984). 6141972 10.1016/0012-1606(84)90172-6

[b25] Peulve P., Laquerriere A., Paresy M., Hemet J. & Tadie M. Establishment of adult rat Schwann cell cultures: effect of b-FGF, alpha-MSH, NGF, PDGF, and TGF-beta on cell cycle. Experimental cell research 214, 543–550, doi: 10.1006/excr.1994.1292 (1994). 7925648

[b26] Manent J., Oguievetskaia K., Bayer J., Ratner N. & Giovannini M. Magnetic cell sorting for enriching Schwann cells from adult mouse peripheral nerves. Journal of neuroscience methods 123, 167–173 (2003). 12606065 10.1016/s0165-0270(02)00349-7

[b27] Vroemen M. & Weidner N. Purification of Schwann cells by selection of p75 low affinity nerve growth factor receptor expressing cells from adult peripheral nerve. Journal of neuroscience methods 124, 135–143 (2003). 12706843 10.1016/s0165-0270(02)00382-5

[b28] Mirsky R., Dubois C., Morgan L. & Jessen K. R. 04 and A007-sulfatide antibodies bind to embryonic Schwann cells prior to the appearance of galactocerebroside; regulation of the antigen by axon-Schwann cell signals and cyclic AMP. Development 109, 105–116 (1990). 2170096 10.1242/dev.109.1.105

[b29] Funk D., Fricke C. & Schlosshauer B. Aging Schwann cells *in vitro*. European journal of cell biology 86, 207–219, doi: 10.1016/j.ejcb.2006.12.006 (2007). 17307274

[b30] Haynes L. W. . Diploid and hyperdiploid rat Schwann cell strains displaying negative autoregulation of growth *in vitro* and myelin sheath-formation *in vivo*. Journal of neuroscience methods 52, 119–127 (1994). 7967716 10.1016/0165-0270(94)90120-1

[b31] Tao Y. Isolation and culture of Schwann cells. Methods in molecular biology (Clifton, N.J.) 1018, 93–104, doi: 10.1007/978-1-62703-444-9_9 (2013). 23681620

[b32] Feng G. Y. & Chang X. L. Effect of the cryopreserved Schwann cells on the peripheral nerve regeneration. Zhongguo ying yong sheng li xue za zhi = Zhongguo yingyong shenglixue zazhi = Chinese journal of applied physiology 19, 82–84 (2003). 21207865

[b33] Roth V. Doubling Time Calculator http://www.doubling-time.com/compute.php (2006).

[b34] Monje P. V., Athauda G. & Wood P. M. Protein kinase A-mediated gating of neuregulin-dependent ErbB2-ErbB3 activation underlies the synergistic action of cAMP on Schwann cell proliferation. J Biol Chem 283, 34087–34100 (2008). 18799465 10.1074/jbc.M802318200 PMC2590688

[b35] Monje P. V. . Non-antagonistic relationship between mitogenic factors and cAMP in adult Schwann cell re-differentiation. Glia 57, 947–961 (2009). 19053056 10.1002/glia.20819 PMC2829776

[b36] Bacallao K. & Monje P. V. Requirement of cAMP signaling for Schwann cell differentiation restricts the onset of myelination. PloS one 10, e0116948, doi: 10.1371/journal.pone.0116948 (2015). 25705874 PMC4338006

[b37] Brockes J. P. & Raff M. C. Studies on cultured rat Schwann cells. In vitro 15, 772–778 (1979). 230150 10.1007/BF02618303

[b38] Kraus A. . Efficacy of various durations of *in vitro* predegeneration on the cell count and purity of rat Schwann-cell cultures. Journal of neurotrauma 27, 197–203, doi: 10.1089/neu.2009.0995 (2010). 19712029

[b39] Jin Y. Q., Liu W., Hong T. H. & Cao Y. Efficient Schwann cell purification by differential cell detachment using multiplex collagenase treatment. Journal of neuroscience methods 170, 140–148, doi: 10.1016/j.jneumeth.2008.01.003 (2008). 18295342

[b40] Hedayatpour A. . A method for isolation and cultivation of adult Schwann cells for nerve conduit. Archives of Iranian medicine 10, 474–480, doi: 07104/aim.0010 (2007). 17903052

[b41] Komiyama T. . A novel technique to isolate adult Schwann cells for an artificial nerve conduit. Journal of neuroscience methods 122, 195–200 (2003). 12573478 10.1016/s0165-0270(02)00320-5

[b42] Kaewkhaw R., Scutt A. M. & Haycock J. W. Integrated culture and purification of rat Schwann cells from freshly isolated adult tissue. Nature protocols 7, 1996–2004, doi: 10.1038/nprot.2012.118 (2012). 23060244

[b43] Wang H. B., Wang X. P., Zhong S. Z. & Shen Z. L. Novel method for culturing Schwann cells from adult mouse sciatic nerve *in vitro*. Molecular medicine reports 7, 449–453, doi: 10.3892/mmr.2012.1177 (2013). 23152081

[b44] Jirsova K., Sodaar P., Mandys V. & Bar P. R. Cold jet: a method to obtain pure Schwann cell cultures without the need for cytotoxic, apoptosis-inducing drug treatment. Journal of neuroscience methods 78, 133–137 (1997). 9497009 10.1016/s0165-0270(97)00146-5

[b45] Haastert K., Seef P., Stein V. M., Tipold A. & Grothe C. A new cell culture protocol for enrichment and genetic modification of adult canine Schwann cells suitable for peripheral nerve tissue engineering. Research in veterinary science 87, 140–142, doi: 10.1016/j.rvsc.2009.01.001 (2009). 19232653

[b46] van Neerven S. G. . Schwann cell migration and neurite outgrowth are influenced by media conditioned by epineurial fibroblasts. Neuroscience 252, 144–153, doi: 10.1016/j.neuroscience.2013.08.009 (2013). 23954802

[b47] Zhang Z. . Fibroblast-derived tenascin-C promotes Schwann cell migration through beta1-integrin dependent pathway during peripheral nerve regeneration. Glia 64, 374–385, doi: 10.1002/glia.22934 (2016). 26497118

[b48] Dreesmann L., Mittnacht U., Lietz M. & Schlosshauer B. Nerve fibroblast impact on Schwann cell behavior. European journal of cell biology 88, 285–300, doi: 10.1016/j.ejcb.2009.01.001 (2009). 19246119

[b49] Saberi H. . Treatment of chronic thoracic spinal cord injury patients with autologous Schwann cell transplantation: an interim report on safety considerations and possible outcomes. Neuroscience letters 443, 46–50, doi: 10.1016/j.neulet.2008.07.041 (2008). 18662744

[b50] Saberi H. . Safety of intramedullary Schwann cell transplantation for postrehabilitation spinal cord injuries: 2-year follow-up of 33 cases. Journal of neurosurgery. Spine 15, 515–525, doi: 10.3171/2011.6.spine10917 (2011). 21800956

[b51] Zhou X. H. . Transplantation of autologous activated Schwann cells in the treatment of spinal cord injury: six cases, more than five years of follow-up. Cell transplantation 21 Suppl 1, S39–S47, doi: 10.3727/096368912x633752 (2012). 22507679

[b52] Guest J., Santamaria A. J. & Benavides F. D. Clinical translation of autologous Schwann cell transplantation for the treatment of spinal cord injury. Current opinion in organ transplantation 18, 682–689, doi: 10.1097/mot.0000000000000026 (2013). 24220051 PMC3864173

[b53] Li J. & Lepski G. Cell transplantation for spinal cord injury: a systematic review. Biomed Res Int 2013, 786475, doi: 10.1155/2013/786475 (2013). 23484157 PMC3581246

[b54] Bunge M. B. Efficacy of Schwann Cell (SC) transplantation for spinal cord repair is improved with combinatorial strategies. The Journal of physiology , doi: 10.1113/jp271531 (2016). PMC4929312 26876753

[b55] Pasquini M. C. . Comparative Outcomes of Donor Graft CD34(+) Selection and Immune Suppressive Therapy As Graft-Versus-Host Disease Prophylaxis for Patients With Acute Myeloid Leukemia in Complete Remission Undergoing HLA-Matched Sibling Allogeneic Hematopoietic Cell Transplantation. Journal of Clinical Oncology 30, 3194–3201, doi: 10.1200/jco.2012.41.7071 (2012). 22869882 PMC3434978

[b56] Salic A. & Mitchison T. J. A chemical method for fast and sensitive detection of DNA synthesis *in vivo*. Proceedings of the National Academy of Sciences of the United States of America 105, 2415–2420, doi: 10.1073/pnas.0712168105 (2008). 18272492 PMC2268151

