# Supplementary material for: A rapid and versatile method for the isolation, purification and cryogenic storage of Schwann cells from adult rodent nerves
Source: Sci Rep. 2016 Aug 23;6:31781. doi: 10.1038/srep31781 (PMC4994039; doi:10.1038/srep31781)
Supplement: Supplementary Information [file srep31781-s1.pdf]

SUPPLEMENTARY INFORMATION

A rapid and versatile method for the isolation, purification and cryogenic storage of Schwann cells from adult rodent nerves

Natalia D. Andersen[1], Shruthi Srinivas[1], Gonzalo Piñero[1,2] and Paula V. Monje[1]*

[1]The Miami Project to Cure Paralysis and Department of Neurological Surgery, [1]University of Miami Miller School of Medicine. Miami, Florida 33136, USA.

[2]Instituto de Química y Fisicoquímica Biológicas (IQUIFIB), Universidad de Buenos Aires. CONICET. Buenos Aires, Argentina.

*Address correspondence to: Paula V. Monje, 1095 NW 14th Terrace, Miami, FL 33136. Phone: 305-243-8259. Fax: 305-243-3923; E-mail: pmonje@miami.edu

Supplementary figures

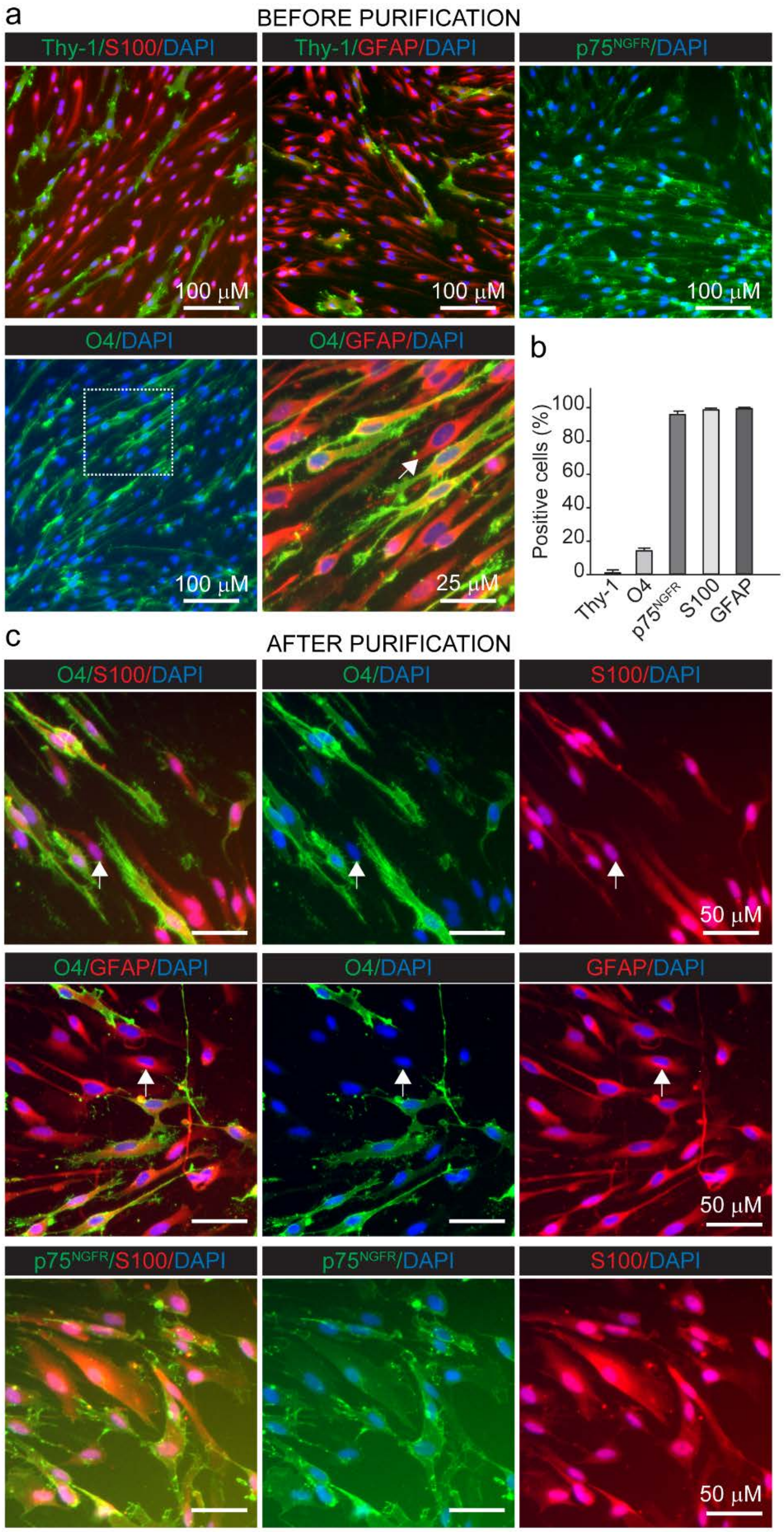

Supplementary Figure S1. Characterization of cultures of rat postnatal SCs before and after purification via negative MACS using Thy-1 immunolabeling. (a) Fluorescence microscopy analysis of postnatal cell cultures before purification. Low magnification images are shown to reveal the extent of fibroblast contamination. Fibroblasts were discriminated from SCs on the basis of their typical Thy-1 positive, $p75^{NGFR}$ negative phenotype (top panels). Whereas the levels of expression of S100, GFAP and $p75^{NGFR}$ were relatively homogeneous across the SC population (upper panels), the levels of expression of O4 varied significantly in individual cells (lower panels). The heterogeneity of O4 expression is more clearly displayed in the higher magnification image shown in the bottom right. Note the presence of SCs that express high levels of GFAP but do not express detectable levels of O4, as indicated by the arrow. **(b-c)** Fluorescence microscopy analysis of postnatal cell cultures after purification. Quantitative analysis (b) and representative images (c) of the resultant cell suspensions from the eluted fraction highly enriched in SCs. Note that $p75^{NGFR}$, S100 and GFAP are expressed in virtually the entire SC population. Resembling the parent postnatal SC cultures (a), a remarkable proportion (~80%) of the purified SCs (b-c) failed to express O4 on their surface despite exhibiting high levels of S100, GFAP, and $p75^{NGFR}$ expression. The images shown in c aim to illustrate the heterogeneity of O4 expression in the purified postnatal cells. Examples of O4 negative SCs are denoted by the arrows (c). These results justify the reliability of using S100, GFAP and $p75^{NGFR}$ rather than O4 for fluorescence microscopy analysis of postnatal SCs prior to and after purification.
